# Carbon Nanotube Wearable Sensors for Health Diagnostics

**DOI:** 10.3390/s21175847

**Published:** 2021-08-30

**Authors:** Monika Rdest, Dawid Janas

**Affiliations:** 1 Department of Materials Science and Metallurgy, University of Cambridge, 27 Charles Babbage Rd., Cambridge CB3 0FS, UK; Monika.Rdest@gmail.com; 2 Department of Organic Chemistry, Bioorganic Chemistry and Biotechnology, Silesian University of Technology, B. Krzywoustego 4, 44-100 Gliwice, Poland

**Keywords:** carbon nanotube wearables, health diagnostics, sensors

## Abstract

This perspective article highlights a recent surge of interest in the application of textiles containing carbon nanotube (CNT) sensors for human health monitoring. Modern life puts more and more pressure on humans, which translates into an increased number of various health disorders. Unfortunately, this effect either decreases the quality of life or shortens it prematurely. A possible solution to this problem is to employ sensors to monitor various body functions and indicate an upcoming disease likelihood at its early stage. A broad spectrum of materials is currently under investigation for this purpose, some of which already entered the market. One of the most promising materials in this field are CNTs. They are flexible and of high electrical conductivity, which can be modulated upon several forms of stimulation. The article begins with an illustration of techniques for how wearable sensors can be built from them. Then, their application potential for tracking various health parameters is presented. Finally, the article ends with a summary of this field’s progress and a vision of the key directions to domesticate this concept.

## 1. Introduction

Three decades have just passed since the (re)discovery of carbon nanotubes [1,2]. Over this time, the interest in these materials has remained at an impressive level thanks to the unique properties offered by these nanostructures. CNTs exhibit remarkable electrical [3,4,5] (high current-carrying capacity, exceptional enhancement of conductivity of composites at low filler loadings), biological [6,7,8,9,10] (antibacterial properties, drug delivery capabilities), thermal [11,12,13] (excellent thermal conductivity), optical [14,15,16] (wide and tunable light absorption/emission spectrum), photocatalytic [17,18] (substantial applicability in pollutant photodecomposition), and mechanical [19,20,21] (extraordinary tensile strength) characteristics, so they are an excellent platform for innovation. What once was a scientific curiosity is a market area of considerable size now, with the annual production of CNTs exceeding thousands of tons. According to Research and Markets, the CNT market is predicted to grow to 9.84 billion U.S. dollars by 2023 from 4.55 billion USD in 2018, translating to a Compound Annual Growth Rate of 16.7% [22]. At this point, as envisioned by Japan’s Yano Research Institute Ltd., the production capacity for CNTs should reach ca. 4000 tons [23]. The progress is driven by an increased application of these materials in coatings [24,25], composites [26,27,28], electronics [29,30,31], and energy-storage systems [32,33,34]. One of the areas, experiencing a considerable increase in the use of CNTs is health diagnostics.

The rapid progress in the miniaturization of electronic devices enables convenient and detailed health monitoring. One can already buy wearable sensors off the shelf embedded inside smartwatches and bands. Such devices can gather and analyze the data day and night to improve the well-being of the owners. For example, they can monitor parameters, such as heartbeat [35,36] and blood oxygen saturation [37,38,39], and track sleep patterns [40,41]. Because such sensors are lightweight and require low power input, it is expected that the next generation of these appliances will be developed as a part of the smart garment. Many wearable prototypes have recently been validated in the scientific literature, which brings hope that this milestone will be reached in the upcoming future. A considerable part of these e-textiles has been prepared with nanocarbon or other nanomaterials at their heart [42,43,44].

**Figure 1:**
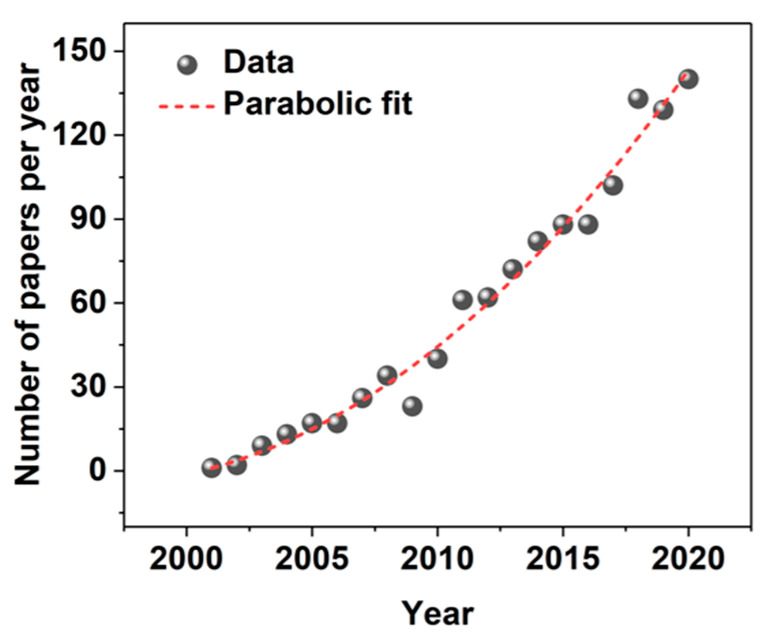
The number of papers per year indexed by the Scopus database under the terms “carbon nanotube*” and “textiles” as of the 19 July 2021.

Carbon nanostructures, such as CNTs or graphene, are ideal for this application because they are flexible [45,46,47], lightweight [48,49,50], highly electrically conducting [51,52], and sensitive to a wide spectrum of stimuli [53,54,55,56,57,58,59], including biomolecules [60,61,62,63,64]. Moreover, their implementation is very welcome, as the typical materials used for wearable sensors nowadays are rigid, which inconveniences the wearer [65], and of inferior mechanical characteristics, thereby making these devices prone to failure upon stretching or bending. In light of the preceding, it is not surprising that the interest in employing CNTs in smart textiles has steadily and rapidly increased over the past years (Figure 1).

Recently, several reviews emerged demonstrating the potential of nanomaterials, such as transition metal dichalcogenides or MXenes [66,67,68], for sensing. However, the field lacks a comprehensive summary of how CNTs produced in large amounts can serve in this area. In this perspective article, the most representative advances in the area of wearable sensors for health monitoring using CNTs exclusively are presented. The paper starts with a description of the primary methods of formation of such materials. These techniques are then evaluated to present the fabrication routes, of which commercial implementation is most feasible. Subsequently, several applications for such innovative materials are highlighted, paying particular focus to their technology readiness level. Finally, the article is concluded with a future outlook, which anticipates the upcoming breakthroughs and suggests research directions that should receive the most immediate attention to catalyze the development on this front.

## 2. Formation of Wearable Sensors from CNTs

**Figure 2:**
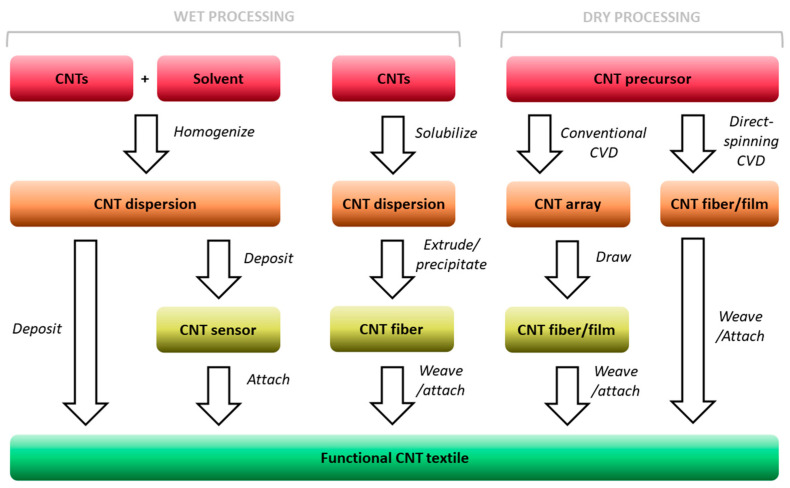
Main-stream routes of formation of functional CNT-based textiles for health monitoring.

Nanocarbon-based textiles are first assembled from individual CNT building blocks for the material to serve the described purpose. Many methods have been devised to carry out such a transformation [69], and they are is summarized in Figure 2. Two approach routes can be discerned: those which involve a liquid medium or the processing techniques conducted in the dry state entirely.

In the former case, the first goal is to “solubilize” the CNTs in a medium capable of overcoming van der Waals interactions between the individual tubes. Although these interactions are weak, the sheer number of them makes this task challenging. A solution to this problem is to employ a surface-active compound, which can facilitate establishing stronger interactions between the CNTs and the solvent rather than themselves.

For water-based solutions, all the classes of surfactants can be employed: anionic (e.g., sodium dodecylbenzene sulfonate [70], sodium cholate [71]), cationic (e.g., cetyltrimethylammonium bromide [72], dodecyltrimethylammonium bromide [73]), nonionic (e.g., Triton X-100 [74], Pluronic series [75]), or amphoteric (e.g., Amber 4001 [76], sodium lauroamphoacetate [77]). On the other hand, organic media typically employ conjugated polymers as surfactants [59], which, as recently discovered, may also exhibit selectivity towards a dispersion of CNTs of a particular structure or electronic character. For instance, poly(N-decyl-2,7-carbazole) was found to disperse almost exclusively semiconducting, single-walled CNTs (SWCNTs) [78], while copolymers containing carbazole and binaphthol moieties recognize monochiral SWCNTs of particular handedness [79].

As an alternative, to make the CNTs much more water-dispersible, one may exercise oxidation, but functional groups on the surface often deteriorate the properties of the material [80,81]. Many reports show that such chemical modification may considerably decrease the capabilities of CNTs to transport charge [82,83,84] or transfer stress [85,86,87]. Thus, physical modification of the material by the incorporation of surface-active chemicals is often engaged. However, a simple combination of CNTs with surfactants is insufficient to make a dispersion from them. Thus, either of the two forms of agitation are typically employed to promote the encapsulation of CNTs with surfactants: sonication [88] and shear mixing [89].

**Figure 3:**
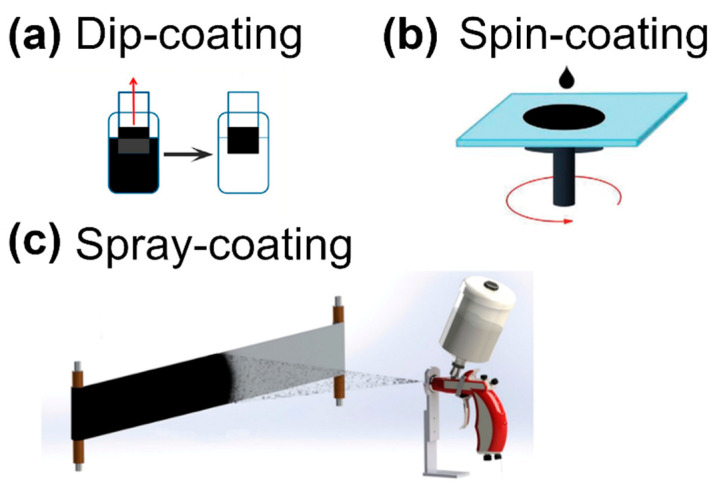
Methods to transform CNT dispersions into coatings and self-standing films without employing porous membranes or conductive substrates. (**a**) Dip-coating reproduced with permission from [94], Copyright the American Chemical Society (2012); (**b**) spin-coating reproduced with permission from [102], Copyright The Authors (2016); (**c**) spray-coating reproduced with permission from [103], Copyright AIP Publishing Ltd. (2014).

After the dispersion is made, an arsenal of techniques is available to transform the suspended CNTs into a macroscopic ensemble in the form of a coating to be integrated with the textile [69]. Since some textiles may be incompatible with particular solvents, a self-standing sensor can be made on a different substrate and then attached to the fabric in the second step. These methods include dip-coating (Figure 3a—repetitive immersion of the substrate into the CNT dispersion) [90,91,92,93,94], spin-coating (Figure 3b—deposition of the CNT dispersion onto a rotating substrate) [95,96,97], and spray-coating (Figure 3c—use of compressed gas to coat the substrate with CNT aerosol) [98] (both spinning and spraying even out the thickness of the CNT coating on the substrate). To achieve the same effect, one can also use vacuum filtration [99,100,101]. In this approach, a CNT dispersion is passed through a porous membrane under reduced pressure.

Due to the substantial aspect ratios of CNTs, which makes their properties highly anisotropic, the CNTs are trapped on the filter while the solvent permeates to the receiving flask. Lastly, a common method to deposit CNTs relies on the fact that the dispersed material can migrate through an electric field (especially when CNTs are enveloped in micelles made from arranged molecules of ionic surfactants, facilitating mobility in the electrolyte). In such a case, the process is referred to as electrophoresis, and a conductive substrate is required for the deposition [104,105,106]. Both of these techniques give rise to the formation of CNT sensors, which are then integrated with an appropriate fabric to create a smart textile. The advantage of the protocols described above is that the CNT powder necessary to make the dispersion is available commercially in large quantities, so they are scalable. Furthermore, the processes are also simple to conduct and free of dangerous chemicals.

**Figure 4:**
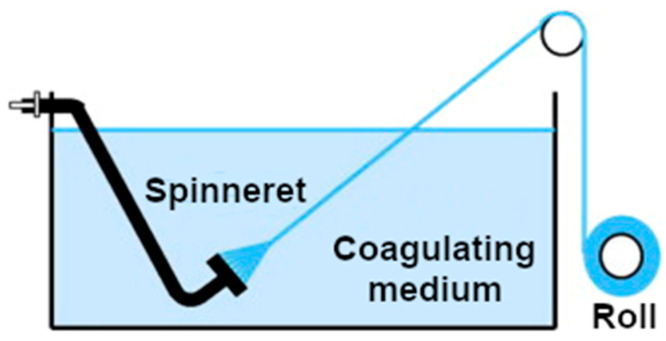
The spinning of CNT fibers modified and reproduced with permission from [114], Copyright Elsevier (2014).

The second type of wet techniques, which can give a functional CNT textile, involves their ensembles in the form of fibers (to learn more about the synthesis and properties of CNT fibers, readers are advised to refer to dedicated reviews on this topic [107,108]). In brief, this method also requires solubilization of the CNTs. One can either use surfactants as described above [109,110] or engage superacids, such as concentrated chlorosulfonic acid, 100% H_2_SO_4_, oleum, etc. [111,112,113], to create liquid-crystalline dispersion of CNTs. The dispersion is then extruded through a bath, where it is precipitated out in a controlled way to obtain a CNT fiber. The fiber created this way is collected continuously onto a roll, the length of which is virtually unlimited. Such well-conductive fibers can already be obtained from the market by the meter. The described concept is presented in Figure 4.

To obtain a similar effect, it is also possible to use electrospinning. An electric field is applied between a dispenser of CNT dispersion, usually kept in a syringe with a conductive nozzle, and a rotating metal collector [115,116,117,118,119]. A DC voltage of sufficient magnitude (on the order of several kV) between these two electrodes enables one to manufacture CNT fibers, which are gradually deposited on the receiver. The largest disadvantage of this process is the slow pace at which the material can be formed.

**Figure 5:**
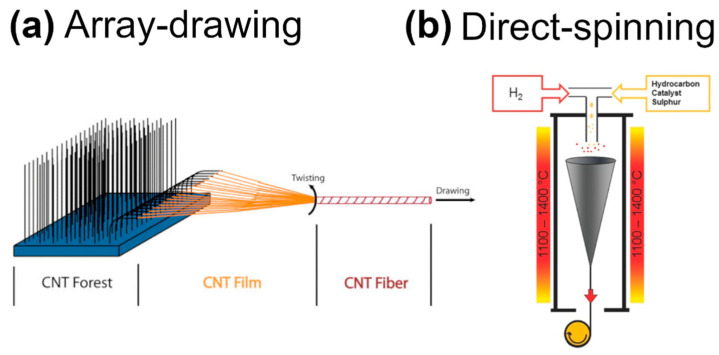
Solid-state formation of CNT fibers by (**a**) array-drawing reproduced with permission from [123], Copyright AIP Publishing LLC (2014); (**b**) direct-spinning modified and reproduced with permission from [107], Copyright The Authors (2014).

There are two more routes that can produce such materials, which, interestingly, obviate the need to employ a liquid medium to disperse CNTs (Figure 5). In the former approach, which is commonly called array-drawing, CNTs are first synthesized by Catalytic Vapor Deposition (CVD) on a substrate in high-temperature conditions [120,121,122]. Then, the material is slowly drawn in the perpendicular direction to the axis of CNT alignment. Due to van der Waals forces, the CNTs adhere to each other well, enabling horizontal alignment of the material [123,124,125,126]. In addition, the formed CNT fibers are often twisted to improve the packing degree, which, in turn, enhances the properties of the material [127,128,129]. It is essential to mention that not CNT arrays are spinnable, which is caused by factors such as the degree of alignment of CNTs in the parent array, the purity, etc. [130,131].

In 2004, it was discovered that this process could be compacted to a single step [132]. When the synthesis is conducted at a higher temperature under hydrogen in a semi-open furnace, CNT fibers are synthesized inside in the form of aerogel. Thus, generated material can be continuously collected on the rotating bobbin to obtain fibers of virtually any length. This approach called direct-spinning can produce kilometers of CNT fibers a day of single-, double-, and multi-walled structure [133].

A yarn produced by either of these methods may be effortlessly woven with a fabric of choice like a regular thread to produce an eTextile. The health-monitoring applications of such yarns and other CNT ensembles described earlier for health monitoring will be presented in the following section.

## 3. The Opportunities Offered by Wearable Sensors from CNTs

It is more and more evident that one needs to combat the negative factors of our daily lives as soon as possible to improve the quality of life and prolong it. That is because the highest chance of a successful medical treatment is shortly after the so-called early detection and then declines at a rate dependent on the disease type. Unfortunately, at this point, the disease symptoms are often not evident at all, or they are so mild that a subject is unaware of their existence. Ideally, to detect these disease indicators as many as possible, health parameters should be monitored constantly and sensitively.

**Figure 6:**
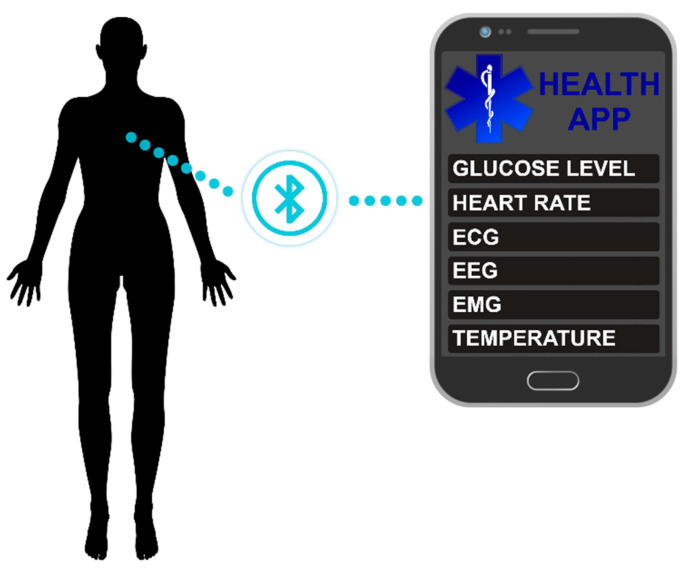
A selection of possible applications of CNTs sensors in smart textiles for health monitoring.

The discovery of nanomaterials and CNTs, in particular, opened new opportunities for this field, as they enable recording of the bioelectrical signals without inconveniencing people too much. This approach was validated by monitoring various health parameters, such as temperature, heart rate, and glucose level in the blood (Figure 6). Furthermore, textile-compatible CNT sensors may be used to record electrocardiography (ECG), electroencephalography (EEG), and electromyography (EMG) signals. The following sections highlight the recent advances in these particular areas of development. 

### 3.1. Glucose Level

Monitoring glucose concentration in the blood is one of the most straightforward methods to detect early signs of diabetes. Once a patient is diagnosed with this condition, such a procedure becomes routine, conducted several times a day. However, invasive needle-pinching is not only inconvenient to many people but also requires attention. Furthermore, measurements carried out a few times a day provide discrete data points rather than continuous results. As a consequence, one cannot accurately track the impact of eating habits. Conversely, constant measurement of glucose concentration in blood would enable predicting a potentially critical condition. For instance, high blood sugar may impair the ability of the body to control blood pressure, which may result in fainting [134]. While passing out at home could result in serious injuries, if consciousness is lost during driving, it may bring about critical/fatal consequences. Excess glucose may also damage the kidneys [135] and induce blindness [136].

**Figure 7:**
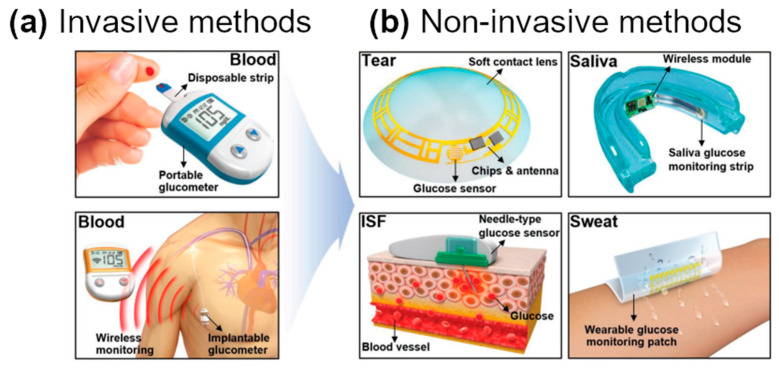
Evolution of glucose sensors using (**a**) invasive and (**b**) non-invasive methods. Modified and reproduced with permission from [137], Copyright Wiley-VCH (2018).

Thus, there is a growing interest in the development of non-invasive glucose sensors [137,138]. Such devices can either be implanted or placed on the skin to gauge the level of glucose in bodily fluids, such as sweat, saliva, tears, or interstitial fluid (Figure 7). Glucose Oxidase (GOx) immobilization on CNTs gives a sensitive electrochemical platform capable of detecting glucose at low concentrations [139,140,141]. Once glucose is contacted with the hybrid material, it is oxidized to gluconic acid, thereby causing changes to the electrochemical characteristics of the sensor. Kang, Park, and Ha showed that a wearable sensor could be constructed from CNTs, which can detect glucose down to 50 μM with a response time of 5 s [142]. The faradic currents generated by the redox process enabled the detection. Although the system did not seem to not monitor glucose concentration directly from the blood, it was nevertheless proven that the implementation of the concept is feasible.

So far, it was shown that glucose levels could be tracked in the venous blood by CNT fibers [143]. Wang and co-workers used CNT fibers to prepare and evaluate CNT fiber-based electrochemical sensors for in-vivo monitoring of various disease biomarkers. Regarding glucose, the sensor’s operating range was 2.5–5 mM, which matched the glucose level in the blood [144]. The real-time monitoring of the level of H_2_O_2_ generated during glucose oxidation facilitated the sensing process. Although the technology-readiness level of these solutions is not at the level of commercial implementation yet, further progress in this area may pave the way towards the utilization of CNT-based textiles for glucose sensing. For instance, the adaptation of microneedles employed in the graphene-based drug-delivery system [145] in a CNT-based glucose sensor could significantly increase its utility.

### 3.2. ECG

**Figure 8:**
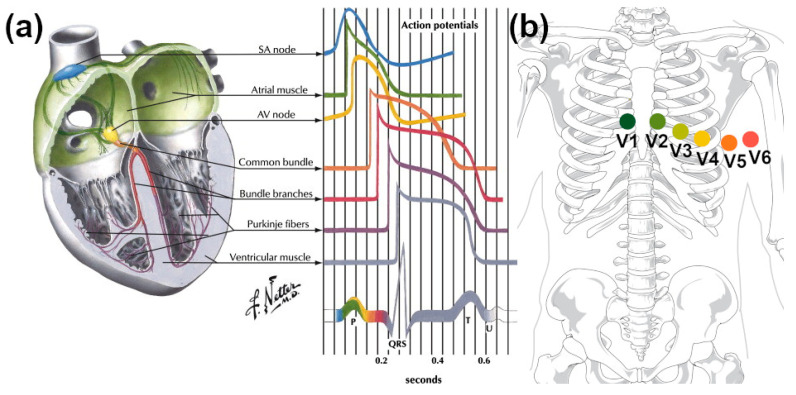
(**a**) Resolution of cardiac signals from individual activities of the heart reproduced with permission from [159], Copyright Elsevier (2014). (**b**) Correct placement of ECG electrodes at the following locations: V1 (4th intercostal space on the right sternum), V2 (4th intercostal space on the left sternum), V3 (equidistant between V2 and V4), V4 (5th intercostal space at the midclavicular line), V5 (anterior axillary line on the same horizontal level as V4), and V6 (mid-axillary line on the same horizontal level as V4 and V5) [160].

Perhaps the most explored area of research in the analyzed field deals with the use of CNTs for electrocardiography [146]. As recently demonstrated by Kolanowska and colleagues, CNTs excel in this field due to their high flexibility, impressive electrical conductivity, and low mass density, which dramatically reduces the device’s weight [147]. Acquisition of ECG signal is conceptually simple, but it requires sufficiently sensitive electrodes of appropriate adhesion to skin to discern all the underlying phenomena. Figure 8a demonstrates major electric features, the shape of which enables studying the condition of a heart. To determine ECG accurately, the electrodes are commonly arranged into 12 leads [148], which are interfaced with a human body at the positions indicated in Figure 8b.

**Figure 9:**
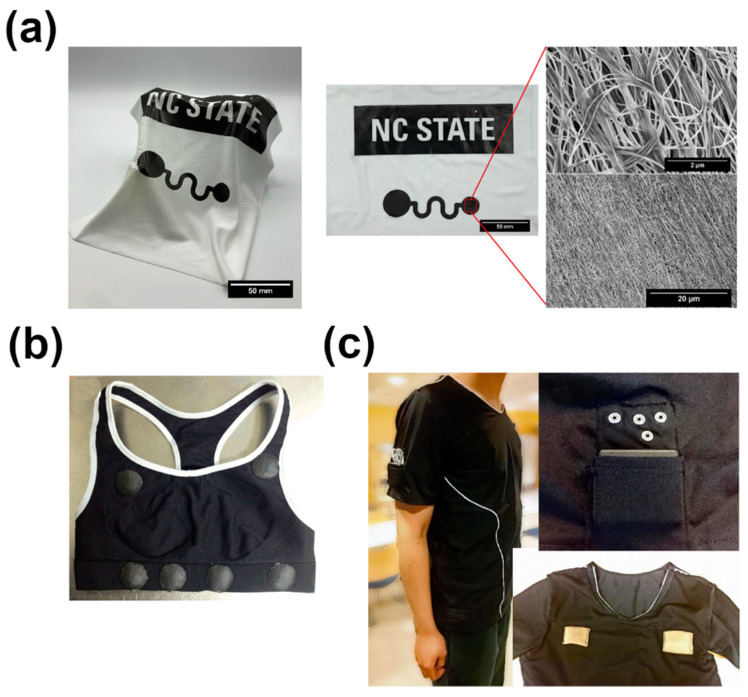
Prototype e-Textiles for ECG measurement: (**a**) t-shirt along with visualization of the microstructure of the material by SEM modified and reproduced with permission from [157], Copyright Elsevier (2020); (**b**) woman’s smart vest modified and reproduced with permission from [147], Copyright Elsevier (2017); (**c**) smart garment with the indication of the placement of textile electrodes and a pocket for a wearable instrument to collect the data modified and reproduced with permission from [158], Copyright The Authors (2019).

An abundance of articles demonstrates how various types of CNTs can be used for ECG acquisition [149,150,151,152,153,154]. Typically, the nanocarbon component is introduced to a flexible polymer matrix, such as polydimethylsiloxane or polyurethane, to make the device convenient to the user and ensure appropriate adhesion to the skin [155,156]. Recently, it was shown that this concept could be taken a step further by incorporating such sensors into textiles [147,157,158] (Figure 9). What is encouraging from the practical point of view is that repeated washing of such textiles showed only a small decrease of performance of 6% in terms of electrical resistance. Regarding the performance, Chi and co-workers showed that a CNT-based sensor can be used for daily monitoring of ECG signals, outperforming the traditional Ag/AgCl electrodes [149]. Despite the impressive progress in this area, we are yet to find out how the proposed e-Textiles for ECG measurement perform in the long term. It is crucial to prove that neither the user nor the smart textile suffers from prolonged use.

### 3.3. EEG

Electroencephalography is employed to record brain activity in real-time by measuring biopotentials through electrodes placed on the scalp [161]. It can be used to detect tumors or track brain activity while the patient is anesthetized. It is also routinely used to study brain disorders such as epilepsy.

The problem with many prototypes for recording EEGs is that the hair on the head negatively affects the signal quality, so, if possible, the measurement is conducted on the forehead instead [162]. As recently summarized by Tseghai and co-workers [163], there is only a handful of reports on the use of textiles for EEG, and none examined CNTs. The most similar material was a textile containing carbon fiber, which showed that a dry electrode built on its base was softer and of lower impedance than conductive rubber [164]. Interestingly, it was observed that the electrode-skin impedance decreases in time as the perspiration accumulation between the skin, and the electrode minimizes it considerably.

However, it does not mean that CNTs cannot be used for this purpose. Awara and co-workers showed that a thin, multi-walled CNT electrode could monitor EEG signals [165]. The prototype electrode was free of metals, which eliminated one of the key problems with the application of conventional Ag/AgCl electrodes. Ag/AgCl blocks X-rays, thereby creating artifacts on Computed Tomography (CT) images during surgery, complicating the procedure. Furthermore, the results showed that CNTs combined with polydimethylsiloxane elastomer could be used for EEG monitoring as well [156]. The concept was verified by measuring signal changes in the alpha band while the sensor was placed on the forehead or earlobe. The proposed fabrication method was simple, fast, and very cost effective. The authors estimated the cost of manufacture of such sensor to be ~1–5 USD/g. Importantly, the CNT/PDMS sensor was very robust, as its properties changed only slightly after subjecting it to 10,000 strain cycles. Lastly, Ruffini and colleagues reported the results of a human trial wherein CNTs were used for the described application [166]. The study showed that (i) CNT-based dry electrodes perform similarly to state-of-the-art wet electrodes, and (ii) there are no side effects such as itching, redness, or irritation six months after their application even when the CNT array was directly exposed to skin. In light of what was said, it is anticipated that the application of CNTs for tracking EEG signals in the form of smart textile materials will emerge soon.

### 3.4. EMG

**Figure 10:**
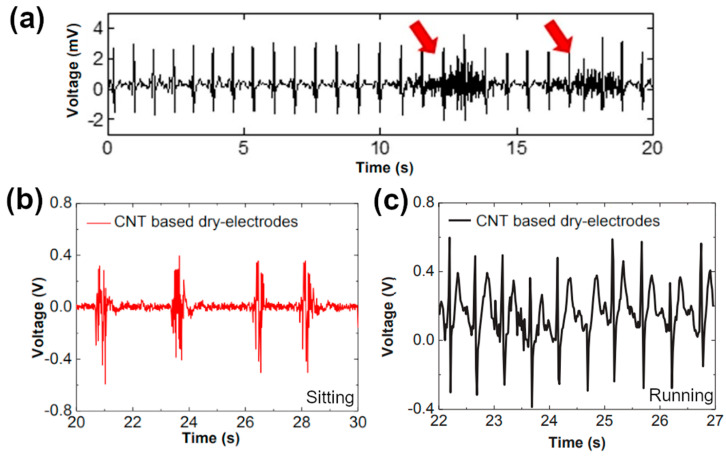
EMG signals acquired using CNT-based electrodes from (**a**) chest modified and reproduced with permission from [168] Copyright The Authors (2014); and (**b**,**c**) forearm modified and reproduced with permission from [169] Copyright The Japan Society of Applied Physics (2018). Panels (**b**,**c**) demonstrate ECG signals while bending the arm during sitting and running, respectively.

Electromyography is a technique used to monitor the well-being of muscles and nerve cells controlling them. The measurement can either be done non-invasively by monitoring the signals on the skin or intramuscularly. Since it was found that nanomaterials can facilitate the detection of EMG signals via the first route, which is much more comfortable, their role on this front has grown considerably over recent times [161,167]. Regarding CNT perspectives, as demonstrated by Lee and co-workers, a combination of CNTs (to do the monitoring) and polydimethylsiloxane (to improve the adhesion) can robustly detect bioelectrical signals even in the presence of wrinkles in the skin. When a subject used the muscles of the chest, EMG signals were discerned by the epidermal sensor (indicated with arrows in the ECG shown in Figure 10a) [168]. There was no need to apply gel to electrically couple skin to the electrodes or glue to improve adhesion, thereby enabling comfortable, continuous, long-term monitoring of the subject.

The electroactivity can be tracked with higher resolution when the variations in myoelectrical potential are gauged away from the chest. Kang and Ha showed that CNT-based wearable electrodes, which can be incorporated in textiles, measure it accurately [169]. As the level of activity was increased from rest (Figure 10b), via walking (not shown) to running (Figure 10c), the signal-to-noise ratio increased substantially. The CNT sensors placed on the forearm showed comparable performance to Ag/AgCl electrodes, which required a conductive gel film. Repeated bending even at a high bend angle did not affect the performance due to the high flexibility of the components.

These advances are significant, as they open new perspectives for people ranging from athletes to the disabled. In the former case, such ECG textile sensors provide means of supervising the muscles’ state while exercising to avoid injury. In the latter case, the solution can be used to evaluate the progress of rehabilitation. Moreover, such CNT sensors can be easily integrated with textiles using various techniques available in the literature for other conductive materials and nanomaterials [170]. For instance, Qi and co-workers showed that yarns made of CNT-coated polyurethane nanofibers can be easily woven to obtain a piezoresistive sensor capable of detection of wrist bending, cheek bulging, swallowing, or respiration [171].

### 3.5. Temperature

The temperature of a human body indirectly informs about several health disorders that may require attention. Furthermore, an excessive amount of heat stored by a body can result in exhaustion and strokes. It is vital for athletes [172] and workers exposed to elevated temperatures, such as miners [173], for whom such conditions may have fatal consequences. The repercussions of the current COVID-19 pandemic could also be minimized if people had means of immediate determination of a temperature rise, leading to earlier self-isolation.

Body temperature is predominantly monitored by wearables operating either on the principles of a thermocouple or by exploiting a phenomenon of a change in electrical resistance with temperature (quantified by the Temperature Coefficient of Resistance—TCR) [174]. Concerning the former, when two junctions composed of dissimilar conductors are exposed to different temperatures, a voltage proportional to a temperature difference is generated. On the other hand, the latter phenomenon capitalizes on the fact that the capabilities of materials to transport charge increase (negative TCR) or decrease (positive TCR) with a temperature rise. In the case of CNTs, the more metallic CNTs are in the material, the higher the probability that the sensor will have a positive TCR at room temperature and above. One may also measure the temperature optically by tracking the angular deformation of waves generated from the sensor reflected from a human body. Still, this approach is less common, as monitoring the change in electrical properties of CNTs is often more convenient.

Blasdel and Monty showed that multi-walled CNTs (6.6 wt.%) deposited on Nylon 6 could resistively determine temperature between 25 °C and 45 °C [175]. Polypyrrole was employed on the top to protect the material from the external environment and improve the electrical percolation between individual CNTs creating the network. The material revealed a negative TCR of −0.228 ± 0.03%/°C. What is important from the practical point of view is that the sensor remained sensitive even after repeated bending and exposure to heating-cooling cycles. No hysteresis was observed. Nevertheless, the operational parameters of the device were affected by the humidity. Therefore, some means of compensation for this effect should always be devised, as making the textile-based sensor impermeable to water vapor would be unwelcome for the skin.

Rosace and co-workers addressed this challenge [176]. The authors combined multi-walled CNTs and cotton to create a wearable sensor to detect changes in humidity and temperature by analyzing the impact of these parameters on surface resistance [176]. CNTs were oxidized, and a number of chemical compounds were added to improve the compatibility with cotton. The resulting eTextile had good mechanical integrity and electrical conductivity. The CNTs adhered to the substrate well and were appropriately interconnected. The parameters mentioned above were monitored, and the designed smart textile showed good sensitivity and reproducibility of results.

**Figure 11:**
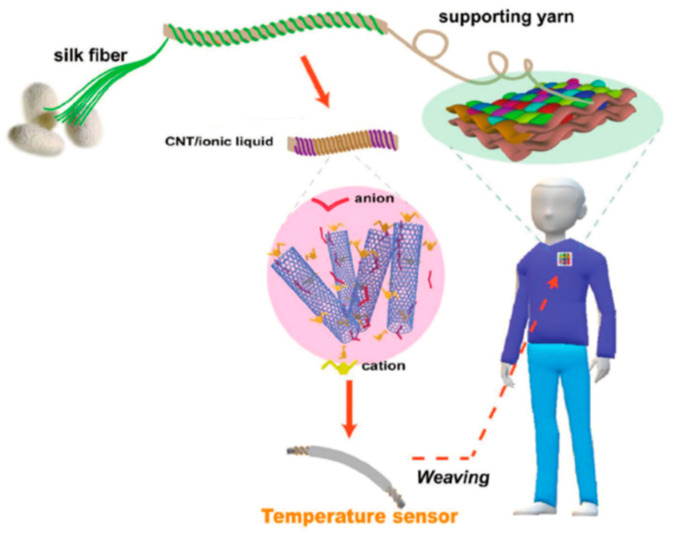
Fabrication of a temperature sensor containing CNTs and silk modified and reproduced with permission from [177], Copyright Wiley-VCH (2019).

Recently, Wu and colleagues reported that similar textiles based on biodegradable/biocompatible materials could be manufactured for a prolonged time [177]. In their contribution, silkworm fiber coiled yarns were combined with CNTs and an ionic liquid to create a temperature-sensitive hybrid fiber, which was then woven into a textile (Figure 11). The obtained material was breathable and durable. Multiple washing cycles did not deteriorate the performance to a significant extent. The presence of 1-Ethyl-3-methylimidazolium bis(trifluoromethylsulfonyl)imide on the surface of CNTs enabled reaching the highest temperature sensitivity among recently reported materials i.e., 1.23%/°C. Due to the appreciable performance, there was no need to employ digital converters or gain amplifiers, the use of which complicates the design of many established temperature sensors. Interestingly, when silk yarns covered with Ag nanowires were additionally woven into the fabric, a combo-sensor able to detect changes in both temperature and pressure was constructed. The reported sensitivity was also high (0.123 kPa^−1^), while the relaxation tie was as short as 0.25 s. Thus, the study demonstrates that a combination of various types of nanomaterials in a smart textile may considerably increase the utility of such materials for health diagnostics.

## 4. Conclusions and Future Outlook

In a relatively short time, the CNT field experienced an impressive amount of progress. What once was an unexpected, soot-like by-product on an electrode is now a multi-billion-dollar industry with the potential to revolutionize many areas of daily life. One area, which has some relation to the whole of humanity and could benefit from the wonders of nanocarbon, is medicine. The ever-increasing pace of life urges the design of new means of monitoring health to stop the growing number of health disorders per capita. To combat this challenge, recently, wearable sensors became the focal point of researchers from every corner of the globe. As this article shows, the majority of the progress has been achieved in the past five years, and the interest from the scientific community in this area keeps increasing. With this in mind, it is likely that the milestones necessary to transfer this technology to our daily lives may be conquered in the upcoming future.

For that to happen, several issues of CNTs should be handled. First of all, despite their merits, the price of the material should further decrease to make the use of the e-Textiles widespread. Often, the best performance is obtained by using single-walled CNTs, which remain much more expensive than multi-walled CNTs. Secondly, there is still a need to gain more control over the structure of the synthesized material. The produced CNTs have a diverse selection of chiralities, which complicates matching the material’s properties with the operational conditions. Having means to synthesize exclusively semiconducting or metallic CNTs, ideally of a pre-defined chirality, would be advantageous. This would simplify the analysis of the signal obtained by their means. Alternatively, rather than focusing on materials science, it may be helpful to adapt machine-learning protocols to handle the complexity of the system.

Furthermore, it is crucial to ensure the biocompatibility of the developed smart textiles having nanocarbon at their heart. CNTs after the synthesis contain residual metallic catalyst, various hydrocarbons, and different forms of (nano)carbon. These components should not be disregarded from consideration. We need to develop ways to purify the material more effectively. One also needs to take into account the recent legislative transition from the Medical Device Directive (MDD) to the Medical Device Regulation (MDR). The new law takes much more precautionary measures for nanomaterial applications in medical devices, so it is of high importance to focus on these issues. Consequently, the long-term use of such e-Textiles should be studied thoroughly to analyze a possible influence of prolonged exposure to CNTs. This is important to ensure the durability of such materials and guarantee that the CNTs stay embedded in the fabric, so they do not affect bodily functions negatively. Although these matters will, most probably, impact the time to market, safety of the users is the priority. Lastly, recent research demonstrates that hybridizing more than one type of nanomaterial can significantly broaden the application opportunities. Thus, it is plausible that a multi-nanomaterial textile could have improved precision or detect many health parameters at once. This would be particularly fruitful for personalized medicine and telemedicine. The former would gain from having a sensitive platform to detect health abnormalities, which could be anticipated for someone with a genetic burden. The latter, on the other hand, would capitalize on the easily accessible, large amount of data, which could be analyzed remotely by a doctor.

## Data Availability

Data regarding this are available from the corresponding author upon a reasonable request.
